# Constraints of weathering intensity in different climate zones on coal accumulation environment

**DOI:** 10.1038/s41598-025-92546-z

**Published:** 2025-03-10

**Authors:** Xiaoping Mao, Xiurong Chen, Zhijing Wang, Fan Yang, Shuxian Li, Yuexing Yang

**Affiliations:** 1 China University of Geosciences (Beijing), Beijing, 100083 China; 2 State Energy Key Laboratory for Carbonate Oil and Gas, Beijing, 100084 China; 3 Tsinghua University, Beijing, 100084 China

**Keywords:** Coal accumulation, Primary productivity, Climate zone, Redbed, Evaporite, Geology, Climate sciences

## Abstract

There are currently multiple hypotheses regarding coal accumulation models, each with certain limitations in applicability. This article investigates the relationship between modern sedimentation, soil and climate to analyze the distribution of coal across different geological periods, aiming to explain the coal accumulation environment within a unified theoretical framework. The research concludes that the intensity of weathering in various climate zones is a determining factor influencing coal formation, which also affects the distribution of two other climate-sensitive sedimentary deposits - evaporite and bauxite. These three types of sedimentary minerals can be considered as paleoclimatic indicators: cold and humid, warm and dry, and hot and humid. Furthermore, the impact of temperature on the mineralization of organic matter is significantly greater than that of redox conditions; in general, the mineralization of organic matter in high-temperature environments at mid-to-low latitudes exceeds productivity, resulting in low carbon sequestration rates. The conclusion drawn is that the optimal environment for peat or coal enrichment exists in a cold-humid climate zone characterized by low primary productivity, rather than in a tropical zone with high primary productivity. Additionally, the latitudinal shifts of cold temperate climate-controlled coal distribution, and the sediment types in these three climate zones sufficiently constrain coal-forming environments.

## Introuduction

There are various hypotheses about coal formation^1,2^. These hypotheses are closely tied to paleoclimatic conditions and redox environments. Currently, there is little controversy regarding the impact of paleoclimate and paleoproductivity on coal accumulation. In discussions of coal-forming environments, some scholars have recognized that the distribution of coal is located in high-latitude temperate regions^3–6^In addition, they concurrently acknowledge that efficient peat accumulation can also occur in tropical regions. For instance, due to the location of the North and South China continental plates near the equator during the Paleozoic period, the humid tropical climate facilitated the formation of two sets of thick coal seams during the Late Carboniferous and Early Permian periods^7^. Zhang et al. (1999)^8^, Wang and Li (1998)^9^suggested that the Carboniferous-Permian coals of the North China Plate developed under a tropical rainforest climate. Rees et al. (2002)^10^concluded that the coals of North China extend nearly to 30° north, and model results indicate that these should be regarded as deposits of the tropical rainforest environment. Coal deposits have been used as an indicator of rainforests of the past^4^. Hodgkins et al.^11^found that near-surface low-latitude peat has greater aromatic content than near-surface high-latitude peat, creating a reduced oxidation state and resulting in recalcitrance. This recalcitrance allows peat to persist in the (sub)tropics. Most coals formed from a prolific growth of vegetation in and adjacent to swamps and removed from active clastic environments in low-lying warm and humid regions^12^. Bao et al. (2023)^6^ suggested that, coal records were associated with a median temperature of 25℃ and precipitation of 1300 mm yr^−1^before 250 Ma (in tropical regions). The possible reason for this is that Pteridophytes, with low water transport capacity, were the dominant plants before 250 Ma^13^, and they preferred to grow in the tropics (0°–15°N–S) where climate was warm and humid. Additionally, redox conditions play a crucial role in controlling the formation of peat. A strongly reducing environment with deeper water overlay is most conducive to the accumulation of peat^14^.

Organic rich black shale is also believed to develop in warm and anoxic environments. Schlanger and Jenkyns^15^ believe that the organic carbon rich sediments developed globally during the Aptian-Albian and Cenomanian-Turonian age are products of “Oceanic Anoxic Events” (OAEs); The basis for oceanic anoxic is the discovery of organic rich black shale in many oceans. Walker-Trivett et al.^16^ suggest that the OAE2 event, which was triggered by Kerguelen volcanism, led to the formation of black shale, and was characterized by super-hot-house conditions alongside oceanic anoxia.

However, these conclusions contradict the carbon sequestration laws of modern environmental science. Environmental science believes that hot or warming conditions are not conducive to carbon sequestration^17,18^. Kai et al.^17^ found that after warming, soil microbial activity intensified, leading to a net loss of soil organic carbon. Chen et al.^18^ revealed that soil warming accelerates carbon emissions from alpine grassland soils. Gudasz et al.^19^ argued that as temperature rise, primary productivity indeed escalates; however, the amount of organic carbon burial does not increase correspondingly. Therefore, in-depth exploration of the ancient environment of peat enrichment has significant scientific importance.

Therefore, this study aims focus on modern sedimentation and utilize variations in weathering intensity across different climate zones as a framework to investigate the relationship between coal distribution during various periods and across different latitudes. The objective is to provide a more coherent explanation of coal formation and accumulation environments within a unified theoretical framework.

## Data and methods

**Fig. 1:**
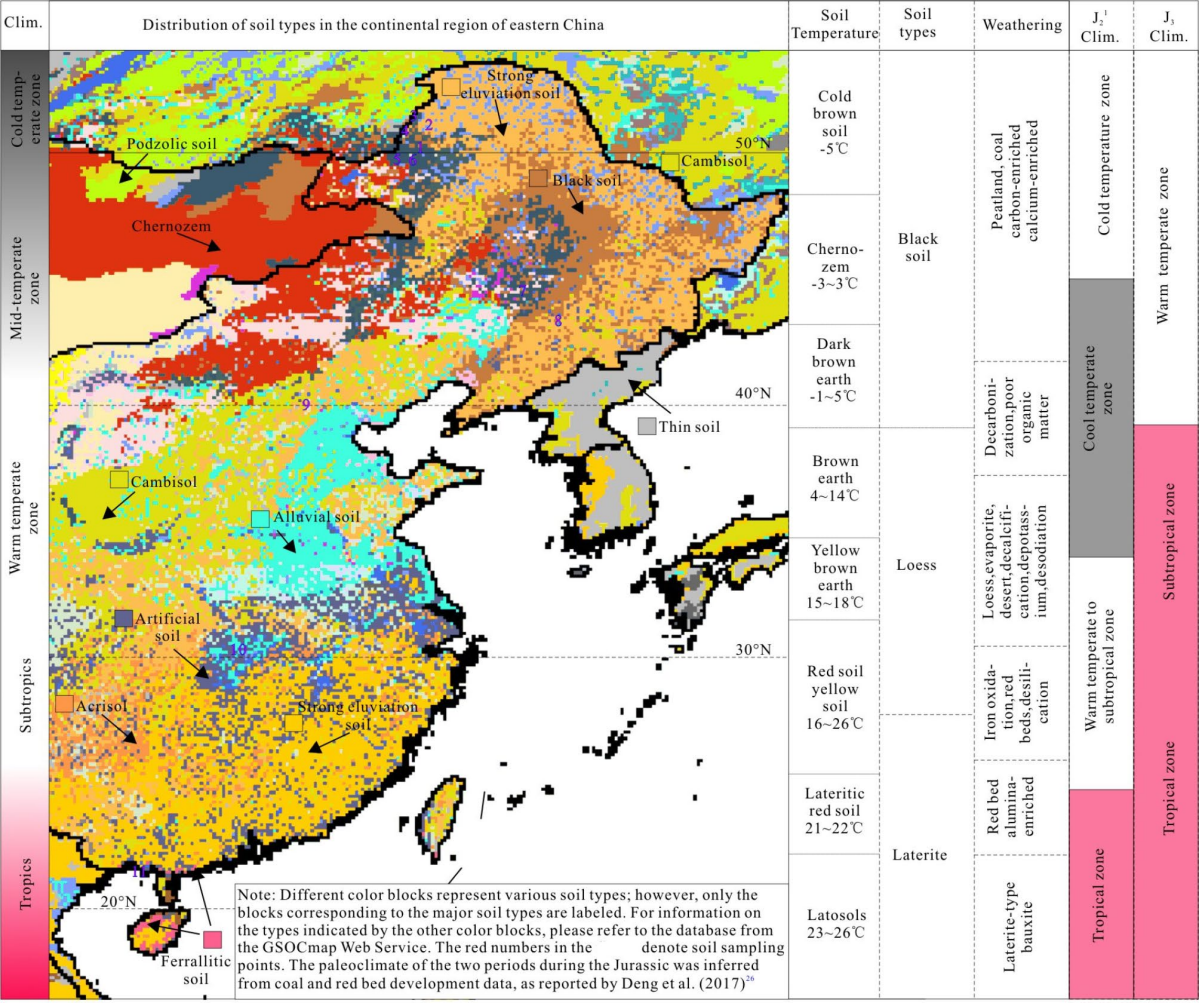
Distribution of weathering conditions and soil types across different climatic zones in eastern China.

The soil sampling method involves the removal of fresh green vegetation and surface litter, followed by the collection of soil cores at a depth of 0–10 cm using a soil drill. Each soil sample, weighing between 200 and 300 g, is then placed into sealed bags for transport. Upon arrival at the laboratory, the samples are processed through a 2 mm sieve, air-dried, and prepared for the determination of soil organic carbon (SOC). Due the depths of the lake and the bay are both less than 6 m, a semi-automatic dredging tool was used to collect surface sediments from 0 to 10 cm depth at the lakebed, with each sample weighing approximately 200–300 g. The pretreatment of these samples and the measurement of total organic carbon (TOC) were conducted at the State Key Laboratory of Shale Gas, China University of Geosciences (Beijing). The carbon isotope and carbon nitrogen ratio of lake sediment samples were measured. Table 1 shows the measured organic carbon content data of soil and wetland surface sediments. The positions of each sampling point are labeled with numbers in Fig. 1. The global soil information mainly comes from the Global SOC map Web Service. Wetland information is derived from the Global Lakes and Wetlands Database (GLWD).

**Table 1:** Sampling locations and organic carbon content test results of soil and lake surface sediment samples.

Sample number	Lithology	Sampling site	TOC (%)	Climatic zone	Sampling time
H1	Soil	Chaoge Shepherd ‘s House, Hailar	4. 54	Cold temperature zone	2023/7/17–22
H4	WSS^1^	The beach of Hailar Forest Park	3. 24		
H5	Soil	Shiwei Port on the slope, Hailar	2. 78		
H7	Soil	Ribbon river Baka, Hailar	3. 44		
H9	Soil	Grassland on the west side of the road in Cuogang Town, Hailar	1. 71		
H10	Soil	Grassland beside the highway travel station from Manzhouli to Hailar	1. 49		
D01	Soil	1 km west of Yushun North Village, Gongzhuling, Jilin	0.88	Mid-temperate zone	
D02	Soil	Same as before	0.91		
D03	Soil	The southwest side of Chaoyang, Huinan, Jilin	1.70		
K01	Soil	The first terrace of Wolong Bay in Kanas Lake, Xinjiang	6.47	Cold temperate zone	2023/6/13
GT01	WSS	Guanting Reservoir in Hebei Province	1.82	Mid-temperate zone	2024/2/18
GT03	WSS		1.17		
GT05	WSS		1.19		
GT07	WSS		1.16		
WHG1T	Soil	Soil of Houguan Lake in Hanyang, Hubei	0.75	Subtropics	2024/4/1
WHG2H	WSS	Lakeside silt of Houguan Lake in Hanyang, Hubei	1.64		
FCG1	BSS^2^	Beihaiwan, Shawan, Fangchenggang, Guangxi	0.90	Tropics	2023/9/30
FCG2	BSS	Shallow sea area of Shawan, Fangchenggang, Guangxi	0.60		

^1^WSS: Wetland surface sediments^2^;BSS: Bay surface sediments.

The research methodology primarily focuses on examining the characteristics of modern sediments, peat and soil across various climatic zones, applying present observations to interpret the past and comparing coal distribution patterns across various geological periods. This approach also consider two other climate-sensitive sedimentary deposits - evaporites, red beds, or bauxite - to constrain coal accumulation environments. This study gathers extensive experimental data on organic matter mineralization within the field of environmental science to elucidate the relationship between primary productivity and organic matter decomposition and mineralization.

## Geological setting

The intensity of weathering varies across different climatic zones and is reflected in the sediments. Consequently, this study focuses on climate-sensitive sediments (lithological indicators of climate) related to climate in both ancient strata and modern deposits. Research on modern climatic environments is primarily concentrated in eastern China, a region characterized by low altitudes, typically not exceeding 200 m, and predominantly flat terrain. The selection of low-altitude plain areas aims to minimize analytical errors that may arise from climatic zone shifts associated with higher altitudes, such as those found in western China. Following the Jurassic period, plate tectonic movements caused western China (longitude < 105°E) to drift northward by approximately 5°−10°. Therefore, the study of climate-sensitive sediments in ancient strata also focuses on basins in eastern China. The climate-sensitive sediments from the Jurassic Period exhibit distinct zonation in eastern China, with red beds developed in the south and carbonaceous shale and coal in the north, which is similar to the distribution of soil types in eastern China.

## Weathering characteristics of different climatic zones

The development of a basin is closely related to the impact of paleoclimate on soil, as the type of soil determines the material source of the basin. Analyzing soils across different climate zones may provide insights into climate-sensitive sediments. Soil can be divided into three categories: black, loess, and red soil, corresponding to three climate zones from north to south in China (Fig. 1):

1. High-latitude cold temperate zone: The soil types in this region consist of frigid brown soil, black soil, chernozem, and dark brown soil. Despite low primary productivity, these soils exhibit low weathering intensity and weak mineralization, resulting in organic- and calcium-rich black soils (Table 1), represented by “cold” and black color.

(2) Mid-Latitude Warm Temperate and Subtropical Zone: The predominant soil types include brown earth, yellow brown soil and yellow soil. The intensity of weathering is significant, resulting in soils that are low in organic matter. Part of organic matter is decomposed, and there is partial loss of calcium, sodium, potassium, and phosphorus, which is represented by the term “warm” and the color white.

(3) Low Latitude Tropical Zone: The soil types include red soil, latosolic red soil and latosol, represented by the term “hot” and the color red. The intensity of weathering in this region is extremely high, leading to significant decomposition of soil organic matter and the loss of essential nutrient elements such as calcium, sodium, potassium and phosphorus are. Even the more resistant weathering silicates undergo decomposition, resulting in the formation of silicic acid, which is subsequently leached away, leaving a relative enrichment of aluminum and iron. Iron is oxidized to trivalent iron, which imparts a red hue, thus giving rise to the well-known red soil^18^. Although this zone exhibits high primary productivity, it is marked by a deficiency in soil organic matter (Table 1).

Obviously, the type of soil has a great relationship with the weathering intensity of different climatic zones. These terrigenous clastic from tropical zone, when transported into lakes and continental shelves by rivers, can also form red beds, bauxite, and iron ore.

## Main controlling factors of peat accumulation

### Statistical characteristics of organic matter enrichment in modern deposits

**Fig. 2:**
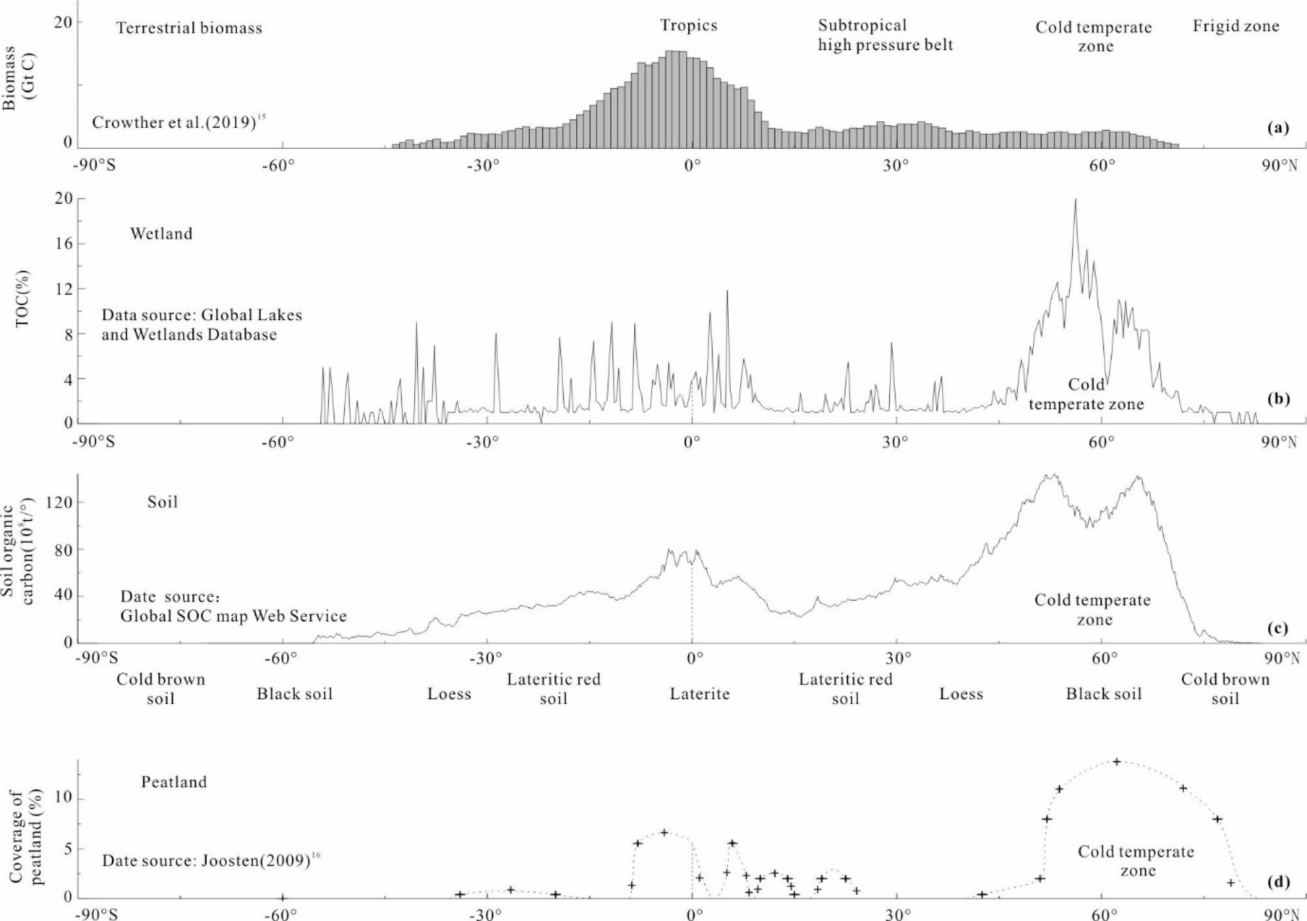
Curve of terrestrial biomass and carbon storage versus latitude. (**a**) Terrestrial biomass(GtC) versus latitude^20^; (**b**) Total organic carbon(TOC) content of wetlands(%) versus latitude; (**c**) Soil organic carbon (SOC) amount per degree (10^8^t/°) versus latitude; (**d**) Peatland F28^21^).

Statistics suggest that, excluding frigid zones, cooler climates may be more conducive to carbon sequestration. As the climate transitions from cold to hot, biomass — an indicator of productivity —increases from the polar regions to the low-latitude equatorial regions (Fig. 2a). In contrast, the carbon sequestration characteristics of soil and wetlands exhibit opposing trend.

The northeastern and northwestern regions of China, as well as Ukraine, Russia, which are known as “global breadbaskets”, are all located in cold temperate zone. Organic matter produced in the summer and autumn seasons is not fully decomposed before winter arrives, and it is preserved in a frozen state below 0℃ in winter. The content of SOC and calcium is very high, forming a famous black soil. From a global perspective, there is also a strong correlation between the TOC content of wetland and latitude which represents varying temperature environments. Wetlands with high TOC content are primarily found in the cold temperate zone, situated between latitudes between 48° and 70° (Fig. 2b). In contrast, wetlands in subtropical and tropical regions at mid-to-low latitudes exhibit lower TOC contents. The relationship between SOC content, peatland coverage, and climatic zones exhibits a similar distribution pattern, with higher values observed in the cold temperature zone and diminished values at mid-to-low latitudes (Fig. 2c and d).

These statistical distribution characteristics indicate that high carbon sequestration rates do not occur in tropical and subtropical warm regions. When discussing the coal deposit environment or the hydrocarbon source rock development, there is a habitual emphasis on high productivity, flourishing flora and fauna in warm conditions, and algae blooms in water bodies^22^, and volcanic ash promoting productivity^23^. Effort are made to demonstrate that the development of coal seams and organic-rich black shale must have corresponded with high primary productivity at that time. Obviously, there is a contradiction between the classic geological theory and these statistical distribution characteristics.

The underlying reason for the contradiction may stem from the differing focuses of coal geology and environmental science. In coal geology, the primary factors controlling coal accumulation are primary productivity and preservation conditions (mainly oxidation-reduction conditions). In contrast, environmental science identifies the controlling factors for carbon sequestration as primary productivity and organic matter mineralization. The latter approach is evidently more comprehensive, as organic matter mineralization is primarily influenced by two factors: temperature and oxidation-reduction conditions. Therefore, it is essential to determine the relative weights of these two factors though experimental methods to address this contradiction.

## Microbial experimental study on the microbial mineralization of organic matter

Experiments on the mineralization of organic matter in environmental science can illustrate the above statistical results.

**Fig. 3:**
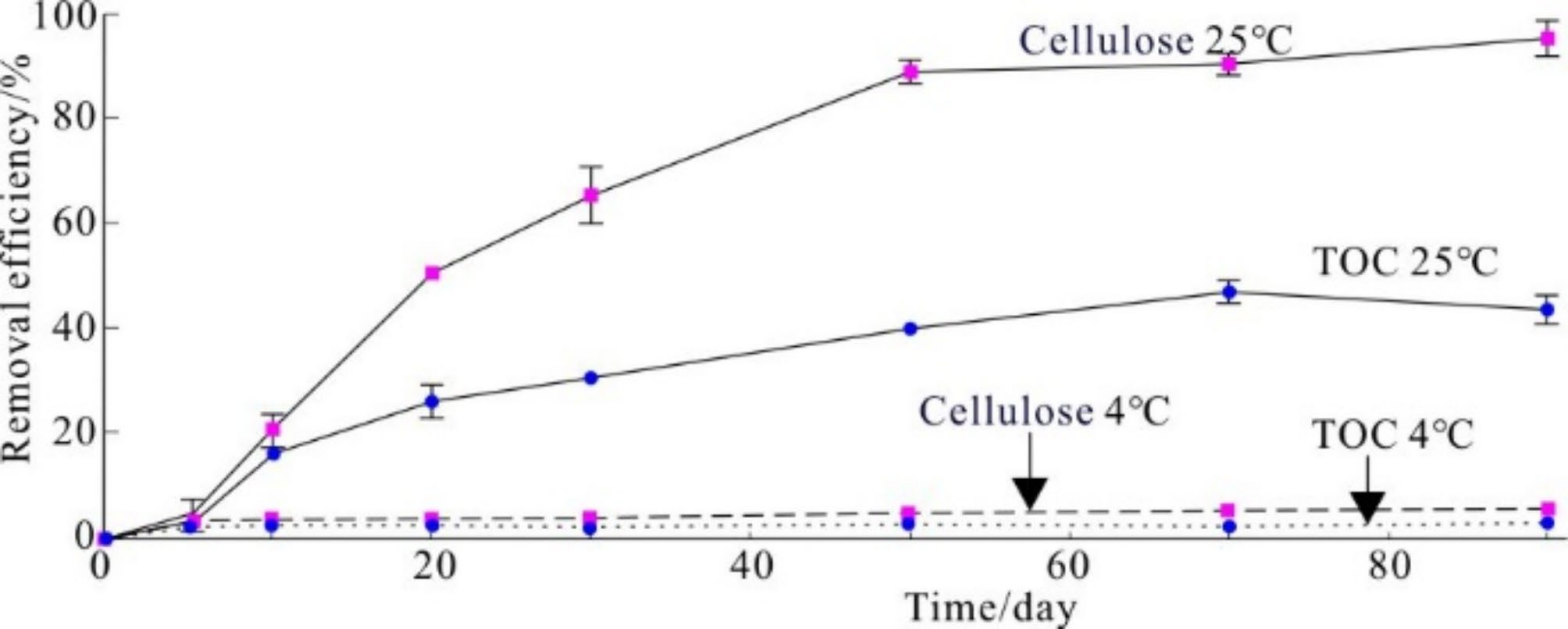
Temporal patterns of cellulose and TOC removal rates of submerged plants in lakes at various ambient temperatures. Data from Chen et al.(2020)^24^.

Chen et al.^24^ experimentally studied the decomposition rates of cellulose and TOC in submerged macrophytes at different temperatures in lake. They found that under 4℃ conditions, there was no significantly degradation of cellulose and TOC in the sediments, with removal rates of only 4.52%±2.54% and 2.01%±1.05%, respectively, over 90 days (one quarter). Whereas in the 25 °C experimental group, the decomposition of Potamogeton malaianus residues resulted in nearly complete breakdown of cellulose, with a TOC removal rate as high as 43.47% ± 2.84% (Fig. 3).

Wei et al.^25^ conducted a study on the decomposition rate of surface sediments over a certain period of time. They set up two groups: one with aeration and one without aeration. The temperature was designed with five levels: 10℃, 15℃, 20℃, 25℃, 30℃. The results showed that, across varying temperatures, the decomposition rate in the oxygen-rich aerated group was only 3–9% higher than that in the anoxic non-aerated group, indicating no significant difference between the two conditions. In the non-aerated group, the decomposition rate at elevated temperature (≥ 20℃) due to anaerobic processes was slightly higher than that observed under oxygen-rich conditions, with the variance remaining within ± 13%. The impact of temperature conditions on decomposition rates is completely different, specifically, the decomposition rate of Chaetoceros at 30℃ is four times greater than that at 10℃ (Table 2).

**Table 2:** Decomposition rate of surface sediment under different temperature and oxidation conditions.

Temperature (°C)	Non-aerated group Anoxia environment(%)	Aeration group Oxygen-enriched environment(%)
10	2. 80	3. 04
15	6. 96	7. 63
20	6. 05	5. 24
25	10. 61	9. 48
30	12. 44	12. 13

(According to Chen et al., 2020^24^).

Gudasz et al.^19^ argued that the higher the water temperature, the higher the mineralization rate of organic carbon and the lower the carbon sequestration rate. Through extensive data fitting, they derived an exponential relation between mineralization rates and temperature, as illustrated in the following equation:

*y* = 10^0. 0362*t*−1. 635^ (1).

In this equation, *y* is the organic carbon mineralization rate in mgC/m^2^/d; *t* is temperature in ℃.

Therefore, among the factors influencing carbon sequestration, the impact of temperature is significantly greater than that of redox conditions. Under low temperature conditions, whether anoxic or oxygen-rich, the mineralization of organic matter is weak, allowing for carbon sequestration. In contrast, in tropical environments characterized by high temperature and high humidity, microbial metabolism remains robust even in the absence of oxygen, leading to significant mineralization. This effectively elucidates the statistical results above derived from modern sediments. As illustrated in Table 1, in the cold temperature zone, TOC can also attain levels of 4–6% (H1, K01) in surface-exposed soils. In the Guanting Reservoir of Hebei Province in the middle temperate zone, the TOC of surface sediments with a depth of 5–6 m is only 1–1.8% (GT01-GT07); the TOC in the shallow sediment of Fangchenggang in tropical Guangxi is only 0.6–0.9% (FC01, FC02).

## Coal accumulation characteristics in different eras

**Fig. 4:**
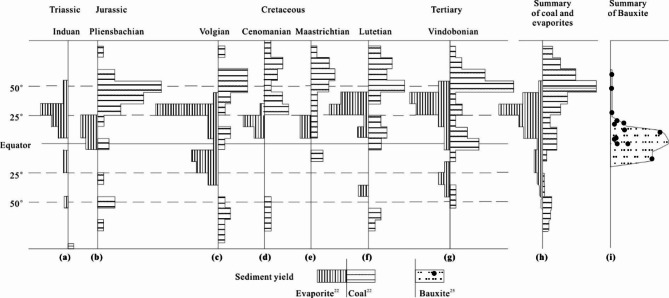
Relationship between the distribution of climate-sensitive sediments and latitude across various geological periods. (**a**)-(**g**) Histograms of the distribution of coals and evaporites by latitude. Data from Parrish et al. (1982)^3^. The length of the bars, which are marked off in increments of 5° indicates the number of deposits in each 10°-wide latitudinal band. (**h**) Distribution of the total abundance of coal and evaporite rocks from (**a**)-(**g**) by latitude. (**i**) Distribution and relative size of major bauxite deposits worldwide versus latitude. Each solid circle represents an individual bauxite deposit, while the solid line serves as the envelope curve for relative reserves (mapping data derived from Zhang et al., 2021^28^).

Analyzing the statistical distribution characteristics of coal in ancient strata can also provide a better understanding of coal accumulation environments. For example, Parrish et al.^3^firstly analyzed the relationship between the distribution of coal and evaporite and latitude in different historical periods, and found that coal is more developed mainly in high latitudes (cold). Warren^26^ and Li et al.^27^ analyzed the relationship between the quantity of evaporite rocks and latitude, and found that evaporite formation was most prevalent in the mid-latitudes subtropical high-pressure zones(warm conditions). Zhang et al.^28^ counted the distribution of bauxite in the world, and found that it is most abundant in tropical regions (hot conditions). These statistical results are basically consistent with modern sedimentation (Fig. 4j). These results show that red beds and evaporite rocks are more likely to develop in high productivity areas at low to mid-latitudes. Only at low temperature in high latitudes, low productivity conditions are more conducive to peat development.

In order to better clarify the characteristics of these three paleoclimatic zones, coal, evaporite, and bauxite are placed on a figure, as shown in Fig. 4. This map includes the distribution of coal rock, evaporite^3^ and lateritic bauxite (data from Zhang et al.^28^) in each period from Triassic to present. Modern wetland data is sourced from the international open wetland database.

In summary, coal, evaporite, and red bed(or bauxite) can be classified as three types of climate - sensitive sediments, representing high-latitude, mid-latitude, and low-latitude climates, or cold-humid, arid, and hot-humid climates, respectively. These three types of climate-sensitive sediments indicate that the optimal development environment for coal is the cold temperate zone.

## The control of zonal shifts of Climatic zones during the jurassic on coal distribution

The global climate is in a state of constant change, leading to variability in climate zones within the same geographical area, which in turn affects the development of geological strata. As a result, coal, which typically forms only in today’s cold temperate zones, may also develop in small quantities near the equator due to climate cooling. Conversely, lateritic bauxite and red beds, usually associated with tropical regions, may also appear in limited amounts within the Arctic Circle as a consequence of climate warming.

**Fig. 5:**
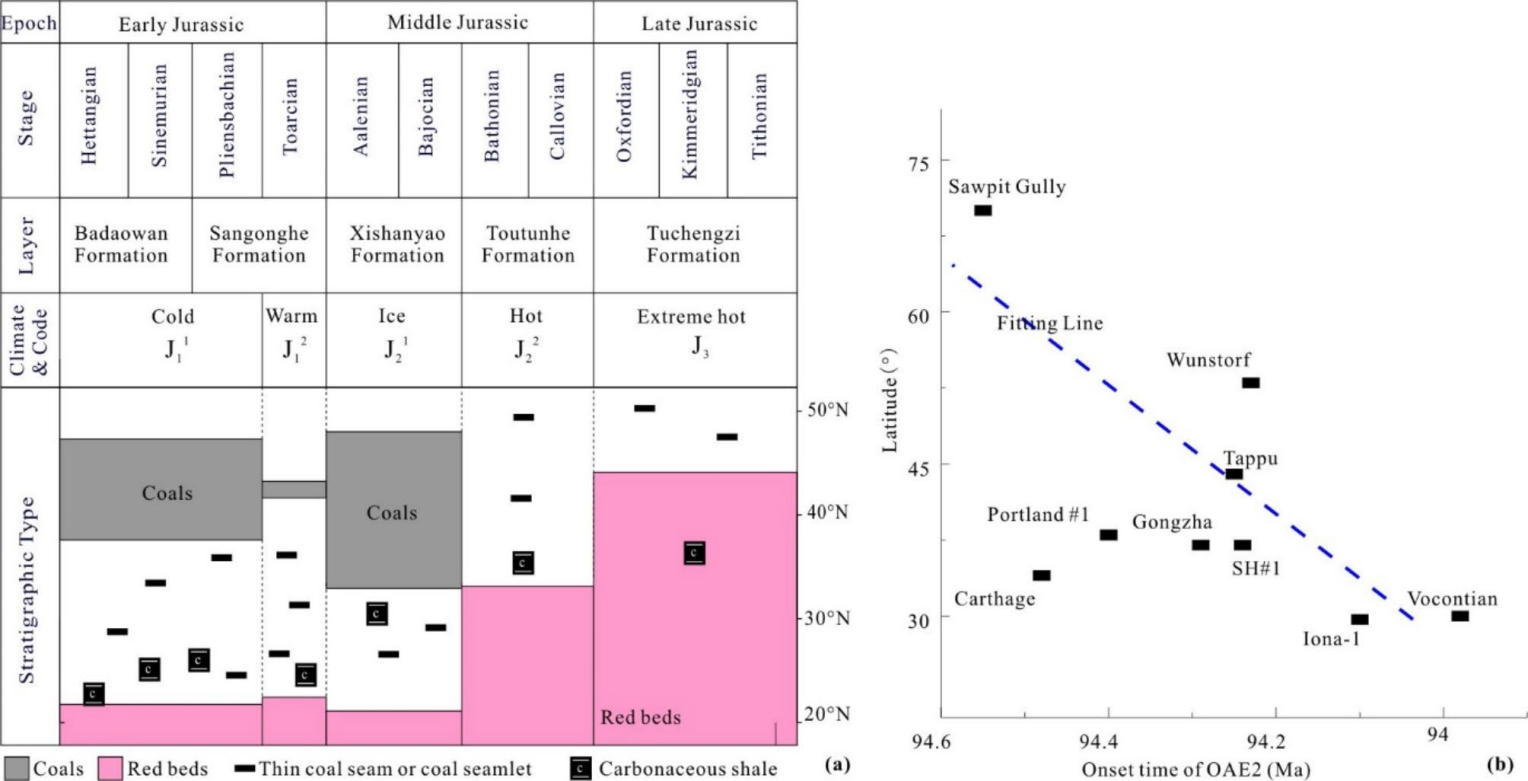
Impact of climate fluctuations on sedimentary rocks. (**a**) Distribution of coal and red beds across various ages of the Jurassic period in the continental region of eastern China. (**b**) Relationship between the onset time of Oceanic Anoxic Event 2 (OAE2) and latitude.

During the Jurassic period, rapid zonal shifts in climatic zones were observed. Deng et al.^29^ categorized the development of the Jurassic strata into five stages, corresponding to the deposition periods of the Badaowan, Sangonghe, Xishanyao, Toutunhe and Tuchengzi Formations. These stages were characterized by cold, warm, frigid, hot and extremely hot climates, respectively, indicating multiple latitudinal shifts in climate zones (Fig. 5a). The paleoclimates for the two extreme stages, the coldest Early Jurassic J_2_^1^ and the hottest Late Jurassic J_3_, are illustrated in the last two columns of Fig. 1. Based on the characteristics of climate-sensitive sediments, we can infer the paleoclimate zones and conclude that the climate during the J_2_^1^ period was colder than the present-day climate, leading to a southward shift of the climate zone. During this time, North China, which is currently classified as a warm temperate zone, was characterized as a cold temperate zone, conducive the development of thick coal seams in the Badaowan Formation. In contrast, during the J_3_ period, the climate zones shifted northward, indicating a warmer climate than that of the present day, which resulted in the cold temperature zone necessary for coal formation migrating northward out of China. As a result, giant thick red beds predominantly developed across nearly the entire Chinese mainland during this period.

Figure 5a illustrates that coal deposits are predominantly found at high latitudes, whereas red beds are characteristic of low latitudes. There is a transitional arid climate zone between these two zones, represented in white. Red and black deposits are mutually exclusive and cannot appear at the same time, as climate serves as the determining factor (data from Deng et al.^29^). Therefore, regions developing red beds at low latitudes are not conducive to coal formation. This leads to the hypothesis that what is currently referred to as ‘tropical peat’ in tropical regions is undergoing further mineralization rather than continuing its development.

Many scholars have analyzed the paleoclimate of the Jurassic coal-forming period and contend that this era likely experienced a cold climate. Dera et al.^30^reconstructed the paleoclimate evolution of the Jurassic period through various means, including oxygen and strontium isotopes and other proxies. The findings reveal a sharp temperature drop in the Badaowan Formation (preceding the Toarcian warm plateau of the Sangonghe Formation) and in the subsequent Xishanyao Formation. The data from the Cleveland Basin in England indicate that there may be a small ice age in the late Pliensbachian (equivalent to the upper part of the Badaowan Formation) of the Early Jurassic Princebachian. The results in Scotland show that the temperature has decreased since Aalenian (equivalent to Xishanyao Formation)^31^. Based on the above, modern peat primarily accumulates in the cold temperate zone, which is located north of 45°N latitude. During these two Jurassic coal-forming phases, the coal-forming zone shifted southward to approximately 31.4°N and 32.35°N, close to the Yangtze River region, indicating that the paleotemperature during this period was likely lower than modern temperatures.

The variations in weathering intensity across different climatic zones, along with the distribution patterns of climate-sensitive sediments, suggest potential deviations in lithostratigraphy, biostratigraphy, and cyclostratigraphy. These deviations are characterized by the presence of the same lithology occurring at different times. For instance, the Cretaceous Oceanic Anoxic Events (OAEs) use the development of black shales as a key indicator. Li et al.^32^ investigated the onset times of OAE2 across various global locations and found significant discrepancies in the initiation of black shale development among different profiles. Based primarily on the data from this study, supplemented by multiple literature sources, a relationship between the onset time of OAE2 and latitude was plotted (Fig. 5b). The figure reveals that black shales initially developed at high latitudes (70°N, 94.55 Ma), gradually progressing southward to mid-latitudes (37°N, 94.29 Ma), and finally to low latitudes (30°N, 94.0 Ma), corresponding to the previously mentioned cold climate of the Aalenian-Bajocian period. This pattern, together with the phenomenon that black shales and coal form in cold temperate zones, indicates a rapid cooling process rather than a calculation error or evidence of atmospheric or oceanic anoxia. As the cold temperature zone shifts north or south at a certain speed, it leaves imprints in the regions it traverses, resulting in the formation of black shale or coal. Although the lithology at different latitudes appears identical, it occurred at different times.

## The genesis of tropical peat

Tropical peat is recognized as an important carbon sink^32^. While this may appear to contradict the main argument of this article, it does not.

Firstly, the volume of tropical peat is relatively small, comprising only one-tenth of the peat found in the Northern Hemisphere^33^, indicating that peat formation in tropical regions is not a common phenomenon.

Secondly, this study focuses on the general rule of the influence of a single factor—climate—on coal accumulation environments, with the aim of minimizing the effects of terrain, altitude, water depth, and other variables. These factors can significantly alter climate zones. For example, although the Qinghai-Tibet Plateau is situated in the subtropical zone, its high altitude causes it to fall under the cold temperate highland zone. China’s richest highland peat deposits are found in this region, with a total volume reaching 48% of the national peat reserves between altitudes of 3,400 to 4,800 m. Additionally, there are 43 extra-large peat deposits, each exceeding 10 million tons, of which 41 are located in the Qinghai-Tibet Plateau and the Yunnan-Guizhou Plateau, collectively representing 95% of the China’s substantial peat reserves^34^. The peat mine in Wangling Town, Binyang County, Guangxi, spans an area of only 0.024 km², with a maximum thickness of 70.86 m. Despite being situated in the subtropics, this mine is located within a low-lying water system that spans nearly 5,000 km². Similarly, the tropical peat collected by Yu et al.^33^ is predominantly found in hilly regions with some elevation or within water catchment areas. The curves presented in Fig. 2b and c indicate a small peak value near the equator. However, this statistical finding does not imply that soils and wetlands in this region are more effective at sequestering carbon. Similar to the Qinghai-Tibet region and Wangling Town, this observation can be attributed to the presence of high-altitude areas or water catchment depressions located near the equator.

**Fig. 6:**
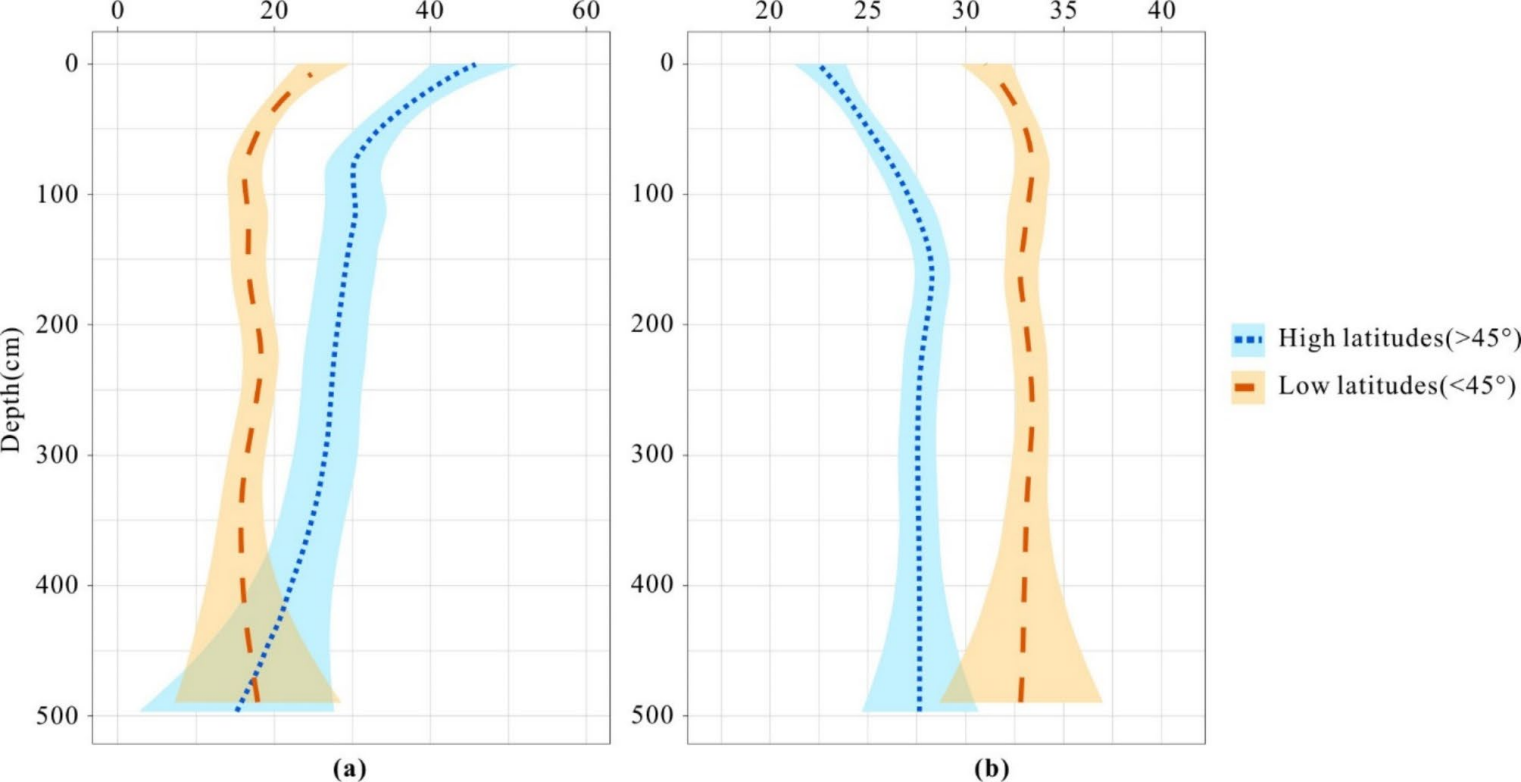
Variations in peat chemistry depth profiles across the latitudinal transect from Hodgkins et al.^11^ (**a**) carbohydrates; (**b**) aromatics.

Third, tropical peat tends to decompose more than it preserves. Hodgkins et al.^11^ proposed that “high-latitude deep peat reservoirs may be stabilized in the face of climate change due to their ultimately lower carbohydrate and higher aromatic composition, similar to tropical peats.” To compare the differences between high-latitude and tropical peat, the authors tested the TOC and aromatic hydrocarbon contents of peat at various depths. The results indicate that the TOC content at a depth of 5 m is basically the same. As the depth decreases, the TOC content in the peat layer at high latitudes increases, more than doubling by the time it reaches the surface. The reduced TOC in tropical peat, in comparison to high-latitude peat, is primarily attributed to the loss of easily decomposed light hydrocarbon components in tropical regions. In the remaining peat layer, the proportion of weathering-resistant aromatic hydrocarbons is 33%, which exceeds the 27% found in high-latitude peat (Fig. 6). Dommain et al.^34^analyzed peatlands in Kalimantan and Sumatra, Indonesia, concluding that equatorial tropical peat, a significant carbon pool, has recently shifted from being a net carbon sink to an important source of atmospheric carbon, and is currently at risk of disappearing. Bai et al.^35^ studied the formation of peat in Chinese subtropical and tropical regions, concluding that while swamp vegetation has high productivity, decomposition process dominating, suppressing peat accumulation. In recent times, no new peat has formed in these areas, instead, only humus has accumulated.

Consequently, determining whether modern tropical peat can be preserved for millions of years to eventually form coal necessitates further in-depth study. Based on the statistics presented in Fig. 5, there is no doubt that thin coal layers, coal seams, and commercially viable coal deposits predominantly occur at high latitudes, suggesting that tropical peat is unlikely to convert into coal over time. Therefore, the hypothesis that coal accumulation occurs in cold and humid climates, as proposed in this article, appears to be universally applicable.

## Conclusion

Based on modern sedimentation and using the idea of “present being a key to the past”, the following conclusions are drawn:

(1) Cold, humid climates in the cold temperate zone or high-altitude cold temperate zones are more conducive to the accumulation of thick peat deposits. The presence of coal seams in warm temperate, subtropical, and tropical regions does not imply that these areas can undergo coal formation within such climatic zones. Rather, it is the result of climatic cooling in these regions, transforming them into cooler temperate zones, which ultimately led to the deposition of coal.

(2) Coal, evaporite, red beds or bauxite represent strata associated with cold-humid climate, warm arid climate, tropical rainforest climate respectively, and they are three types of stable and reliable climate-sensitive sediments.

(3) The mineralization of organic matter is much more sensitive to temperature than to redox conditions.

(4) High carbon sequestration rates are observed in high-latitude regions (excluding frigid zones) characterized by low primary productivity. In contrast, regions with high primary productivity located in low latitudes tend to exhibit lower carbon sequestration rates.

(5) The red-to-black shift from red beds to (black) coal development in strata reflects a climatic shift from warm to cold, while the black-to-red shift indicates a transition from cold to warm climate, independent of whether the environment is anoxic or oxygen-rich.

At last, it is worth mentioning that, coal geology, petroleum geology, and even geology in general may have overemphasized redox conditions. Redox conditions cannot replace the mineralization of organic matter, or in other words, coal geology almost do not take into account the decomposition of organic matter. In higher temperature soil and aquatic environments, mineralization can still occur due to anaerobic bacteria even under hypoxic conditions. Conversely, in low-temperature environments, organic matter can be well-preserved even in oxygen-rich conditions due to poor microbial activity. Therefore, in the future, we should objectively reduce the influence of redox conditions and pay attention to the important influence of climatic zones on peat enrichment.

## Data Availability

All data references and sources used in this manuscript have been shown in the article, and there are no other supplementary data.
